# Central fibrous areas: changes in glomerular vascular pole lesions associated with age and disease

**DOI:** 10.1007/s11255-022-03126-3

**Published:** 2022-01-31

**Authors:** Yukiko Kanetsuna, Kazunari Tanabe, Motoshi Hattori, Kosaku Nitta, Takahito Moriyama, Shigeru Horita, Yutaka Yamaguchi

**Affiliations:** 1 Department of Pathology, International University of Health and Welfare, Atami Hospital, 13-1 higashi-kaigan-cho, Atami, Shizuoka 413-0012 Japan; 2 Department of Urology, Tokyo Women’s Medical University, 8-1 Kawada-cho Shinjuku-ku, Tokyo, 162-8666 Japan; 3 Department of Pediatric Nephrology, Tokyo Women’s Medical University, 8-1 Kawada-cho Shinjuku-ku, Tokyo, 162-8666 Japan; 4 Department of Nephrology, Tokyo Women’s Medical University, 8-1 Kawada-cho Shinjuku-ku, Tokyo, 162-8666 Japan; 5 Kidney Center, Tokyo Women’s Medical University, 8-1 Kawada-cho Shinjuku-ku, Tokyo, 162-8666 Japan; 6 Yamaguchi’s Pathological Laboratory, 1-31-20 Minoridai Matsudo-shi, Chiba, 270-2231 Japan

**Keywords:** Central fibrous area, Aging, Renal donor, IgA nephropathy

## Abstract

**Purpose:**

Central fibrous areas (CFAs) are small, hyalinotic, monotonous nodular areas observed in glomerular vascular pole lesions. We attempted to clarify the relationship between CFA formation and age in healthy kidneys and in those affected by immunoglobulin A (IgA) nephropathy.

**Methods:**

Zero-hour biopsy specimens from living renal donors (135 cases) and IgA nephropathy biopsy specimens (67 cases) were collected retrospectively. We observed each biopsy specimen and determined the total number of glomeruli, total level of glomerulosclerosis, number of observable glomerular vascular poles, number of glomeruli with CFAs, serum creatinine level, and estimated glomerular filtration rate (eGFR). Additionally, we calculated the glomerular sclerosis rate (GSR), vascular pole appearance rate (PAR), and CFA rate (CFAR) to evaluate the relationship between these factors and patient age.

**Results:**

There was a significant negative correlation between patient age and eGFR for both the zero-hour (*p* < 0.0001 in Spearman, *p* = 0.0009 in multiple regression, the same hereafter) and IgA (*p* = 0.0022, *p* = 0.0001) groups. In the zero-hour group, we observed a significant positive correlation between patient age and GSR (*p* = 0.0001, *p* < 0.0001); however, there was no such correlation in the IgA group. In both groups, there was a significant positive correlation between patient age and CFAR (zero-hour group: *p* = 0.0003, *p* = 0.0091, IgA group; *p* < 0.0001, *p* = 0.0004). The slope of the regression line of the IgA group formula was also significantly higher than that of the zero-hour group formula (*p* < 0.01).

**Conclusion:**

These findings indicate that CFA may be a useful indicator of kidney aging, especially in patients with kidney disease caused by IgA nephropathy.

## Introduction

**Fig. 1:**
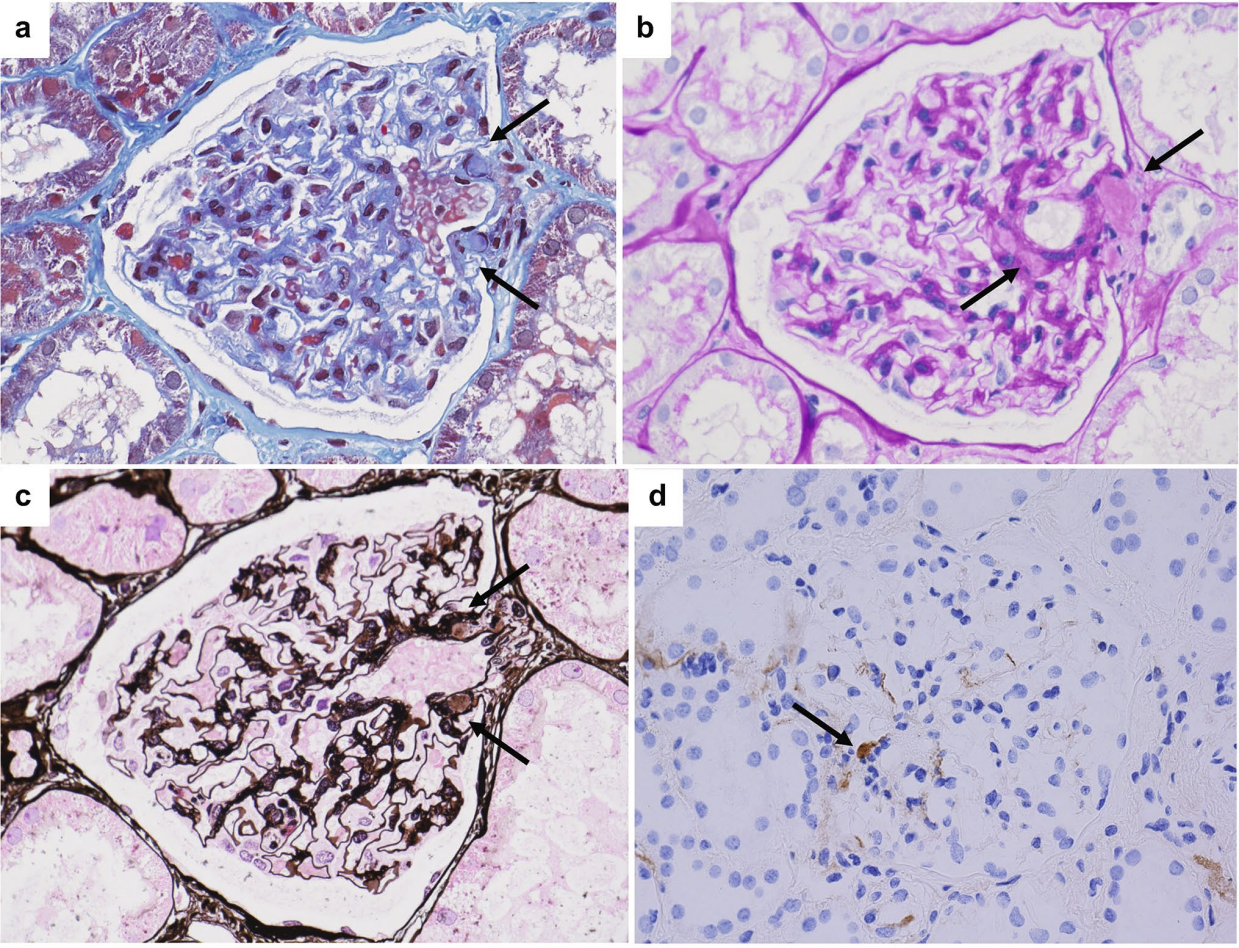
Light microscopic findings of central fibrous areas (CFA) in the same glomeruli (**a**–**c**) and immunoglobulin M (IgM) immunohistochemical staining of another glomeruli (**d**). Small monotonous hyalinotic nodule in a glomerular vascular pole lesion (arrow). The nodule was stained blue by Masson staining (**a**) and exhibited weakly positive periodic acid Schiff (PAS) staining (**d**), negative or weakly positive periodic acid silver-methenamine (PAM) staining (**c**), and immunohistochemically positive IgM staining (arrow) (**d**)

Changes to the vascular poles of the renal glomeruli have been reported much less frequently than changes to other parts of the glomeruli, such as mesangial lesions [1] and those affecting podocytes [2–4] and the glomerular basement membrane [1, 3, 5, 6]. However, small hyalinotic nodular areas have sometimes been observed in glomerular vascular pole lesions. These nodules are monotonous and are stained blue by Masson staining, are weakly positive for periodic acid Schiff (PAS) staining, and can be immunohistochemically positive for immunoglobulin M (IgM) (Fig. 1).

Few reports have described these nodules. Our search identified only one report by Inomata [7] published in 1986, in which the author referred to the lesions as vascular pole deposits (VPD). However, we have observed these nodules in renal histopathological specimens at some frequency. Therefore, we believe that the nodules form as a result of some significant effects on the kidney tissue. In the present study, we defined nodules as central fibrous areas (CFAs).

To date, no statistical evidence for the association between CFA formation and patient age has been reported. Therefore, in this study, we attempted to clarify the relationship between CFA formation and aging using renal biopsy specimens. We also attempted to evaluate the influence of glomerular disease (represented by immunoglobulin A (IgA) nephropathy) on CFA formation. Given that we have observed that CFAs are common in the kidney tissue of older patients, we hypothesized that renal tissue aging promotes CFA formation.

## Materials and methods

Living renal donors from the Department of Urology of Tokyo Women’s Medical University and patients with IgA nephropathy who underwent their first renal biopsy at the Department of Nephrology or Department of Pediatric Nephrology of the same university were biopsied between January 2014 and June 2015. All donors underwent a zero-hour biopsy during donor nephrectomy. All donors and patients with IgA nephropathy with diabetes and/or a glomerular disease other than IgA nephropathy were excluded to eliminate external factors that could affect kidney structure to the greatest extent possible. Patients who were prescribed antihypertensive agents were included if their blood pressure was successfully controlled.

The participants were divided into two groups as follows: the zero-hour group, which consisted of donors without IgA nephropathy, and the IgA group, which included patients with IgA nephropathy. Donors diagnosed with IgA nephropathy when a zero-hour biopsy was performed were also classified into the IgA group.

Information regarding age, sex, serum creatinine (sCr), and estimated glomerular filtration rate (eGFR) were reviewed retrospectively from the patients’ medical records just before their donor operation or renal biopsy. We used eGFR data from patients who were ≥ 18 years old when the renal biopsy was performed because the eGFR calculation formula for children younger than 18 years differs from that for adults, and eGFR had not been calculated for most of the child patients.

### Pathological examinations

We used the prepared specimens from stocks that had previously been clinically diagnosed. Donor zero-hour biopsy specimens were collected via wedge biopsy. Renal biopsy specimens from the Department of Nephrology or the Department of Pediatric Nephrology were collected via needle biopsy.

Biopsy samples were fixed in 10% buffered formaldehyde and embedded in paraffin blocks. The samples were sliced into 2-μm thick sections and stained with hematoxylin–eosin (HE), Masson, elastica-Masson, PAS, and periodic acid silver-methenamine (PAM)-HE. These specimens were observed using a light microscope to examine the number of total glomeruli, total level of glomerulosclerosis, presence of glomeruli with an observable vascular pole, and presence of glomeruli with a CFA in their vascular pole lesion. We determined that a CFA was present when a monotonous nodular lesion was observed in two serial sections of the same glomerulus.

We calculated several values for each case. The glomerular sclerosis rate (GSR) was the rate of the glomerulosclerosis to the total number of glomeruli. CFAs are histopathological findings that can be recognized only in glomeruli with an observable vascular pole. For this reason, the number of glomeruli with an observable vascular pole lesion in each specimen should be the denominator for the numerical evaluation of CFA appearance. We calculated the rates of glomeruli with an observable vascular pole to non-sclerotic glomeruli, then nominated the result as the vascular pole appearance rate (PAR). Then, we calculated rates of glomeruli with CFA to glomeruli with an observable vascular pole lesion in each specimen, and nominated the result as the CFA rate (CFAR).

The formulas for GSR, PAR, and CFAR are as follows: GSR = glomerulosclerosis number/total glomerular number, PAR = number of glomeruli with an observable vascular pole/(total glomeruli – total with glomerulosclerosis), and the CFAR = number of glomeruli with CFA/number of glomeruli with an observable vascular pole.

### Statistical analysis

We calculated the key statistics of each clinical and histopathological parameter for the zero-hour and IgA groups. Continuous variables were expressed as the mean ± standard deviation (SD). To compare data between the zero-hour and IgA groups, we used two-tailed Mann–Whitney *U*-tests except for the male/female ratio, for which the 2 × 2 chi-square test was used.

Spearman’s rank correlation coefficients (two-tailed) and multiple regression analysis were used to evaluate the relationships between patient age and the sCr, eGFR, GSR, and CFAR in each group. However, for the eGFR evaluation, we only used data from patients aged ≥ 18 years for the 135 zero-hour cases and 58 IgA cases. In addition, we evaluated the difference between the zero-hour and IgA groups’ regression curves using covariate analysis.

Statistical significance was set at *p* < 0.05. Statistical analyses were performed using Microsoft Excel version 16 (Microsoft Corporation, USA).

### Ethical considerations

The study was performed in accordance with the 2013 Declaration of Helsinki. Written comprehensive permission was obtained from the patients or the parents of minor patients before the donor operation or renal biopsy. Informed consent was obtained in the form of opt-outs on the website of Tokyo Women’s Medical University. The medical ethics committee of Tokyo Women’s Medical University approved this study protocol (approval no. 3802-R2).

## Results

### Patient and demographic data

**Fig. 2:**
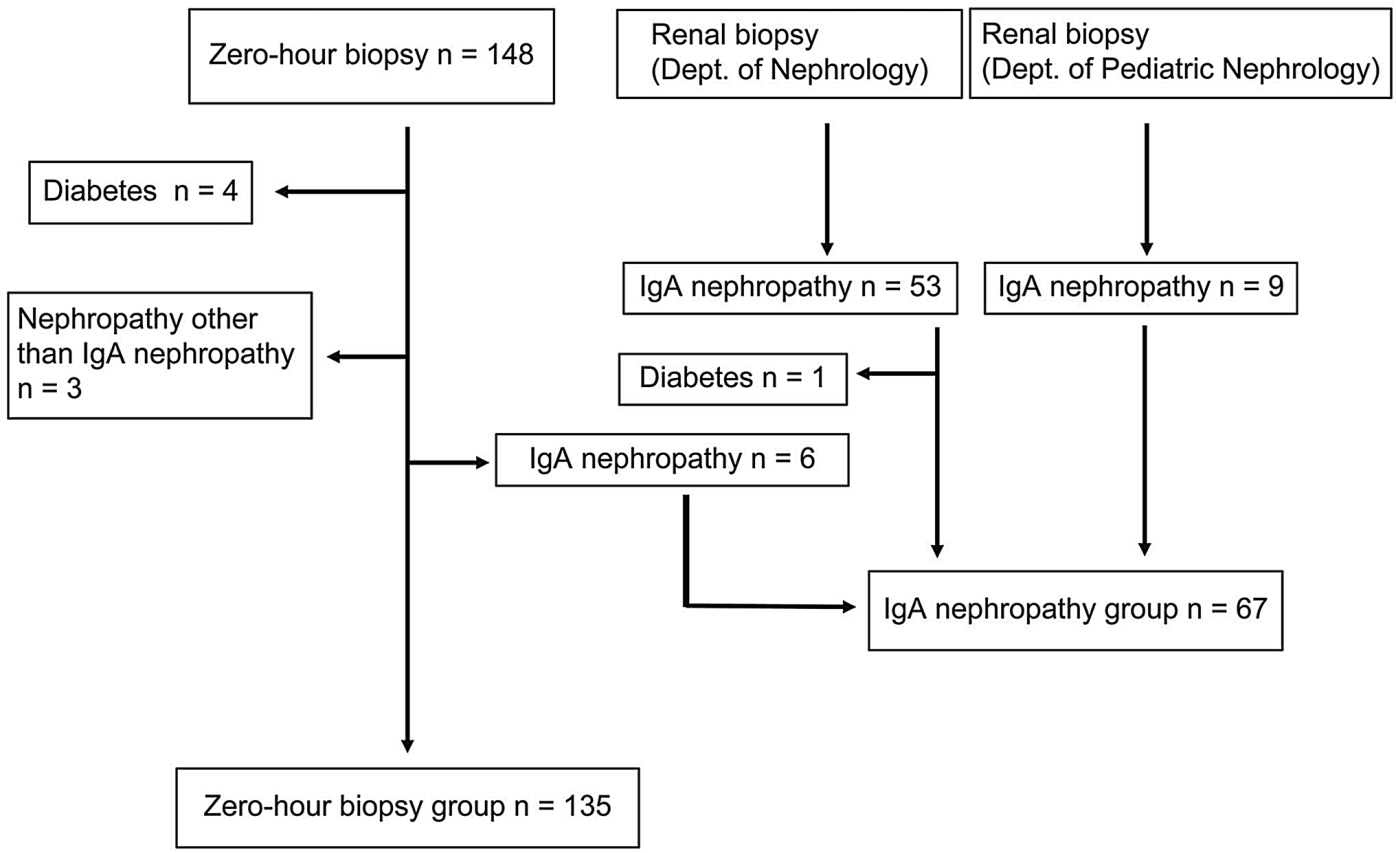
Flow chart of study participant selection

Figure 2 shows the flowchart of participant selection. One-hundred forty-eight living donor nephrectomies with zero-hour biopsies were performed between January 2014 and June 2015. Four donors with diabetes and three donors with glomerular diseases other than IgA nephropathy were excluded from the evaluation. Six donors were diagnosed with IgA nephropathy at their zero-hour biopsies, and these donors were shifted to the IgA nephropathy group. The remaining 135 donors were included in the zero-hour group.

During the same period, 62 patients were diagnosed with IgA nephropathy based on their first renal biopsy (53 from the Department of Nephrology and nine from the Department of Pediatric Nephrology). Patients with IgA nephropathy and diabetes were excluded from the study. The final IgA group consisted of 67 participants: six donors and 61 patients with IgA nephropathy.

Table 1 shows the demographic and clinical characteristics of the study groups. The mean age was 58.3 ± 11.2 years in the zero-hour group and 33.1 ± 13.9 years in the IgA group. The zero-hour group participants were significantly older than the IgA group participants (*p* < 0.001). The male/female ratio was not significantly different between the zero-hour group (male 48.3%) and the IgA group (male 41.8%). The sCr level was higher in the IgA group than in the zero-hour group (0.77 ± 0.25 mg/dL vs. 0.67 ± 0.13 mg/dL, respectively, *p* < 0.001). In contrast, for those aged ≥ 18 years, the eGFR was higher in the IgA group than in the zero-hour group (81.3 ± 18.7 mL/min/1.73m^2^ vs. 79.24 ± 13.71 mL/min/1.73m^2^, respectively, *p* < 0.001).

**Table 1:** Demographic and clinical characteristics of the zero-hour and IgA groups (mean ± SD [range], except for male/female ratio)

	Zero-hour group	IgA group	*p* value
Number of participants	135	67	
Age (mean ± SD)	58.3 ± 11.2 (29–65)	33.1 ± 13.9 (9–64)	< 0.001
Male/female (male %)	44/91 (48.3)	28/39 (41.8)	0.199
Serum creatinine (mg/dl)	0.67 ± 0.13 (0.40–1.11)	0.77 ± 0.25 (0.39–1.75)	< 0.001
eGFR (ml/min/1.73m^2^)	79.24 ± 13.71 (54.3–119.0)	81.3 ± 18.7^a^ (35.5–123.4)	< 0.001

^a^Estimated glomerular filtration rate (eGFR) from patients aged ≥ 18 years (58 patients)

### Renal biopsy findings

Table 2 shows the histopathological characteristics of renal biopsy specimens from both groups. The total number of glomeruli and the total number of glomerulosclerosis cases in each biopsy specimen were significantly higher in the zero-hour group than in the IgA group (*p* < 0.001). GSR and PAR were higher in the IgA group than in the zero-hour group (12.8 ± 12.2%, vs. 10.0 ± 9.35% and 43.2 ± 11.9% vs. 37.3 ± 10.0%, respectively, both *p* < 0.001). However, the overall CFAR was significantly higher in the zero-hour group than in the IgA group (17.4 ± 15.3% vs. 13.1 ± 21.0%, respectively, *p* < 0.001).

**Table 2:** Histopathological characteristics of renal biopsy specimens (mean ± SD [range])

	Zero-hour group	IgA group	*p* value
Total glomerular number	49.7 ± 27.8 (3–165)	19.9 ± 12.8 (4–65)	< 0.001
Global sclerosis number	5.1 ± 5.7 (0–31)	2.3 ± 2.3 (0–9)	< 0.001
GSR (%)	10.0 ± 9.35 (0–50.0)	12.8 ± 12.2 (0–55.6)	< 0.001
Vascular pole number	17.6 ± 11.5 (1–75)	7.7 ± 5.4 (0–26)	< 0.001
PAR (%)	37.3 ± 10.0 (13.3–71.4)	43.2 ± 11.9 (0–75.0)	< 0.001
CFA number per one biopsy specimen	3.0 ± 3.1 (0–21)	1.2 ± 2.5 (0–13)	< 0.001
CFAR (%)	17.4 ± 15.3 (0–76.5)	13.1 ± 21.0 (0–100)	< 0.001

*GSR* glomerular sclerosis rate, *PAR* vascular pole appearance rate, *CFAR* central fibrous area rate

### Relationship between age and clinical and histopathological factors

**Fig. 3:**
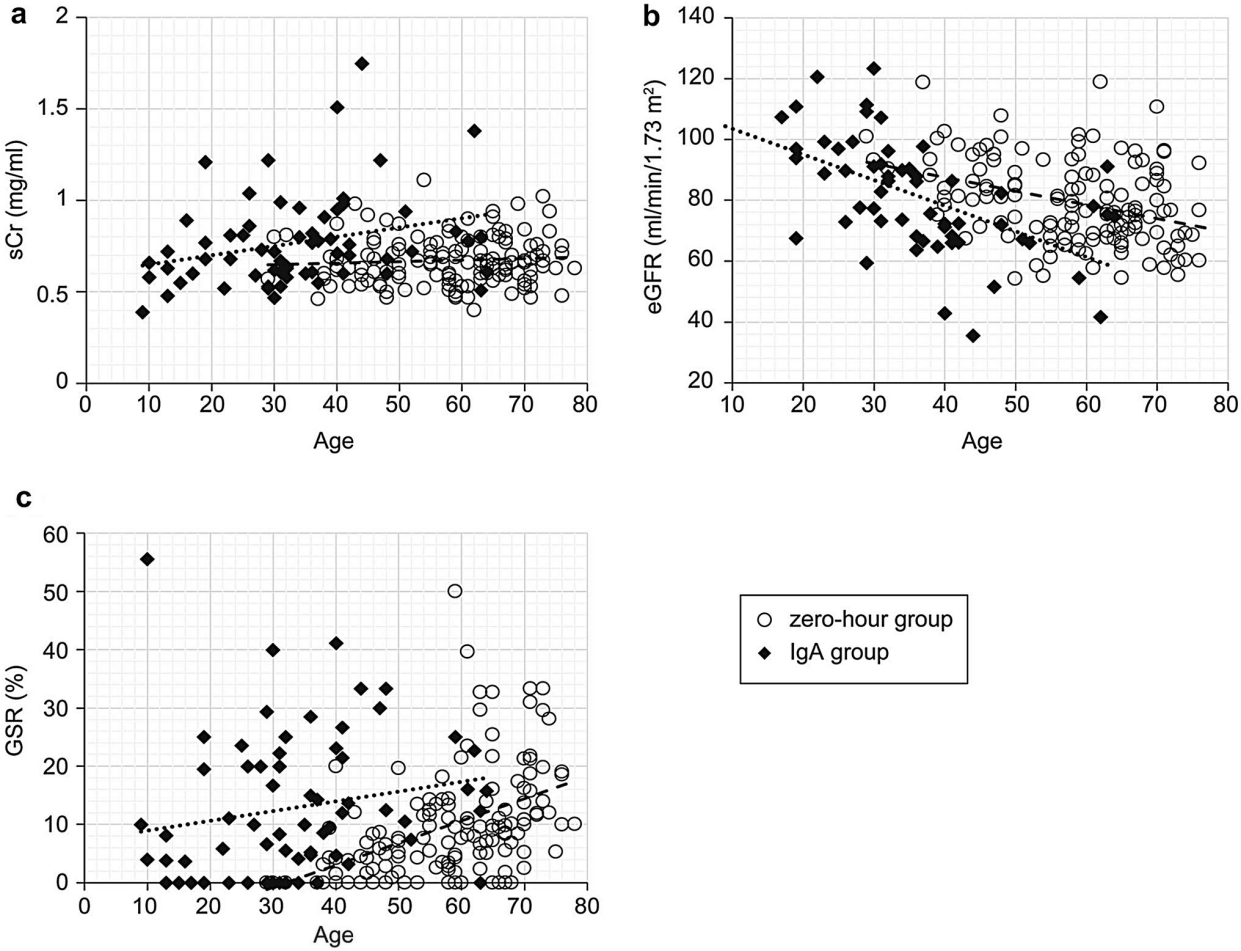
Relationship between patient age and serum creatinine (sCr) (**a**), estimated glomerular filtration rate (eGFR) (**b**), and glomerular sclerosis rate (GSR) (**c**). **a** For both the zero-hour and IgA groups, the relationship between patient age and sCr was unclear. **b** For both the zero-hour and IgA group, there was a significant negative relationship between patient age and eGFR. **c** For the zero-hour group, there was a strong positive correlation between patient age and GSR; however, for the IgA group, there was no significant relationship between patient age and GSR. Open circle: zero-hour group; closed lozenge: IgA group; rough broken line: regression line of zero-hour group; fine broken line: regression line of IgA group

**Fig. 4:**
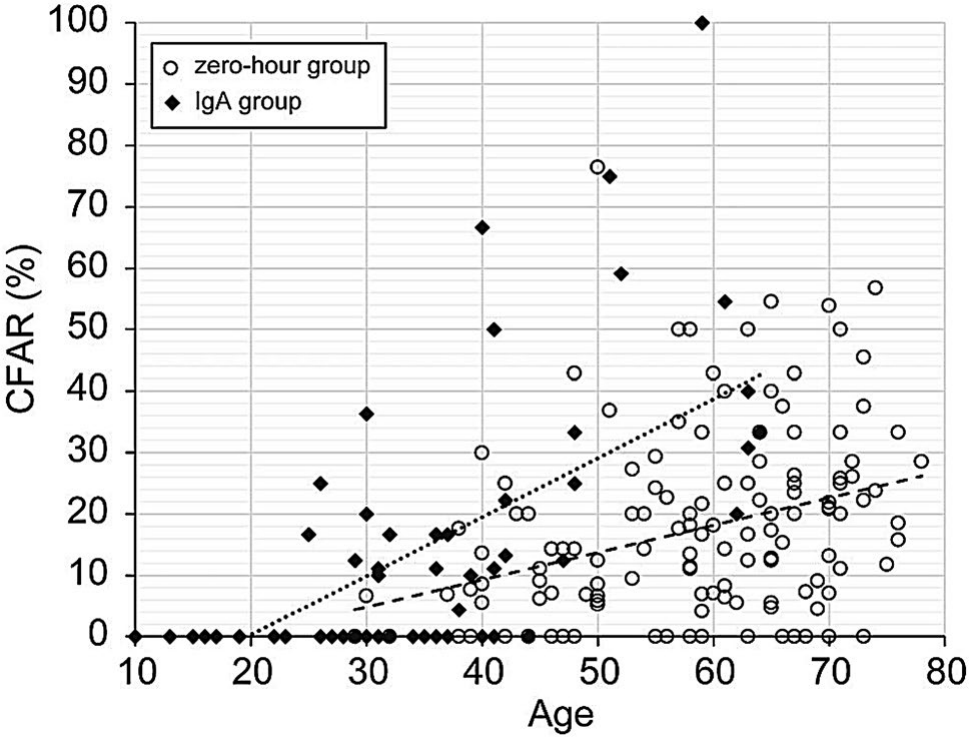
Relationship between patient age and central fibrous area rate (CFAR). For both the zero-hour and IgA groups, there was a significant positive correlation between patient age and CFAR. The inclination of the regression line of the IgA group formula was significantly higher than that of the zero-hour group formula (*p* < 0.01). Open circle: zero-hour group; closed lozenge: IgA group; rough broken line: regression line of zero-hour group; fine broken line: regression line of IgA group

Table 3 shows the results of the correlation analysis between patient age and sCr, eGFR, GSR, and CFAR in the zero-hour and IgA groups. In the zero-hour group, patient age was significantly associated with eGFR, GSR, and CFAR for both the Spearman’s rank correlation coefficient and multiple regression analysis. The relationship between patient age and eGFR was significantly negative, and the relationship between patient age and GSR or CFAR was significantly positive (Figs. 3 and 4). In the IgA group, patient age was significantly associated with eGFR and CFAR. The correlation coefficient between patient age and CFAR was particularly strong (*r* = 0.7751). However, there was no significant correlation between the age of patients with IgA nephropathy and their GSR.

**Table 3:** Results of correlation analysis between patient age and serum creatinine (sCr), estimated glomerular filtration rate (eGFR), glomerular sclerosis rate (GSR), and central fibrous area rate (CFAR)

	Spearman, two-tailed		Multiple regression analysis
	*r* value	*p* value	*p* value
Zero-hour group			
sCr	0.0703	0.4076	0.0391
eGFR	0.6985	< 0.0001	0.0009
GSR	0.7917	0.0001	< 0.0001
CFAR	0.7370	0.0003	0.0091
IgA group			
sCr	0.2731	0.8407	0.0242
eGFR	0.6349	0.0022	0.0001
GSR	0.1669	0.1127	0.9703
CFAR	0.7751	< 0.0001	0.0004

For both the zero-hour and IgA groups, there was a significant relationship between patient age and sCr in the multiple regression analysis, but not in the Spearman’ s rank correlation coefficient analysis.

### Relationship between age and CFAR

Figure 4 shows the relationship between age and the CFAR. The youngest kidneys with CFAs belonged to a 30-year-old patient in the zero-hour group and a 25-year-old patient in the IgA group. As previously described, for both groups, there was a significant positive correlation between patient age and CFAR. In the zero-hour group, kidneys with ≥ 10% CFAR belonged to patients aged > 37 years. However, one 25-year-old patient in the IgA group had a kidney with ≥ 10% CFAR. Overall, the CFAR was significantly higher in the zero-hour group than in the IgA group (Table 2). In the kidneys of patients aged 50–60 years, the CFARs tended to be higher in the IgA group (51.5 ± 26.3%) than in the zero-hour group (18.2 ± 15.7%). However, there were no significant differences between the two groups. We also calculated a regression formula between patient age and CFAR. The formulas were *y* = 0.4444*x* − 8.4871 (*R*^2^ = 0.1058) for the zero-hour group and *y* = 0.9607*x* − 18.949 (*R*^2^ = 0.4019) for the IgA group. The slope of the regression line of the IgA group formula was significantly higher than that of the zero-hour group formula (*p* < 0.01) in the covariate analysis. For this reason, we concluded that CFAR increased with age in both the zero-hour and IgA groups, and that CFAR increased more in the IgA group than in the zero-hour group.

### Relationship between CFAR and clinical and histopathological factors

**Fig. 5:**
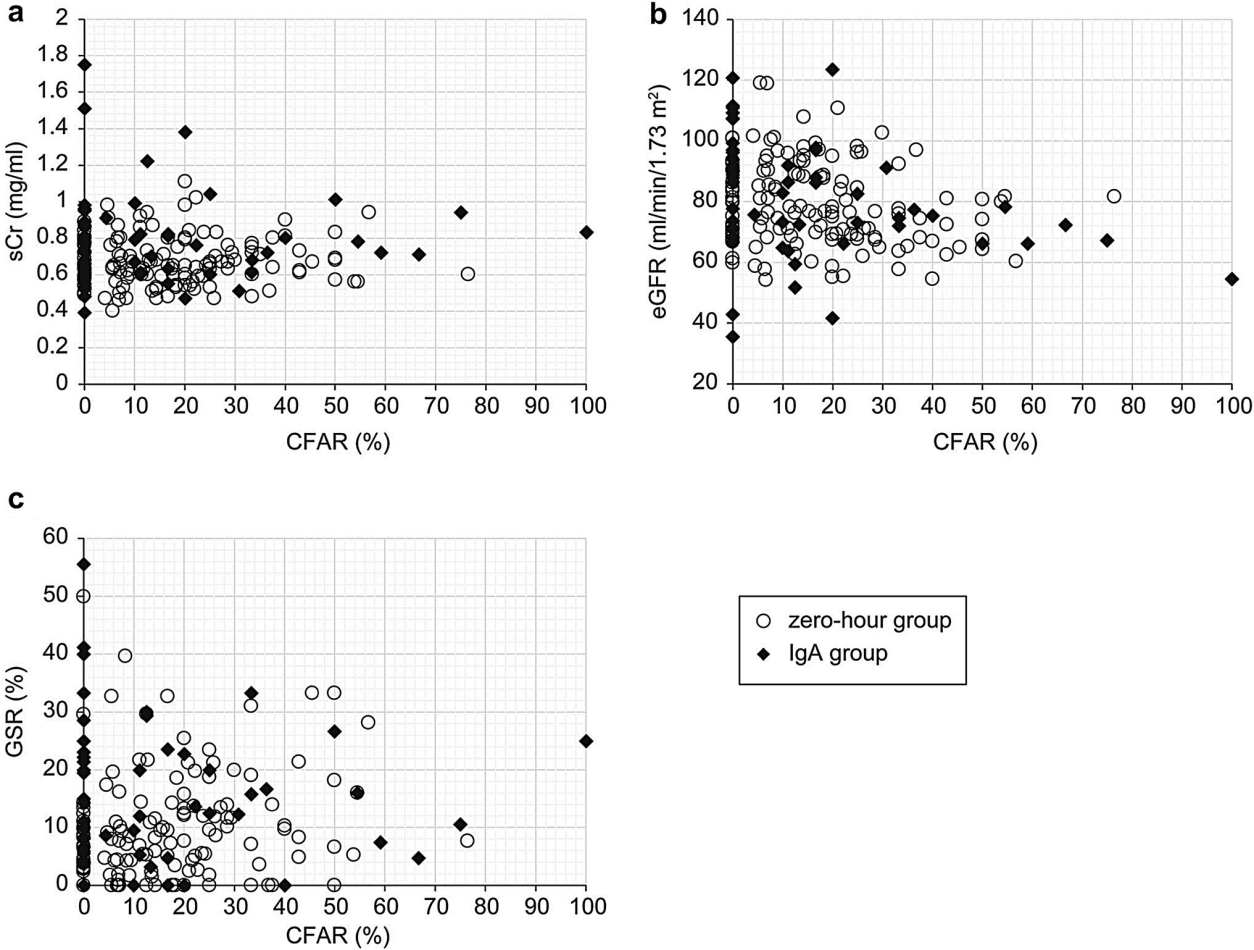
Relationship between central fibrous area rate (CFAR) and serum creatinine (sCr). (**a**), estimated glomerular filtration rate (eGFR) (**b**), and glomerular sclerosis rate (GSR) (**c**). There was no significant relationship between CFAR and sCr, eGFR, or GSR in the zero-hour group. However, in the IgA group, there was a significant relationship between CFAR and sCr, eGFR, and GSR in the Spearman’s rank correlation coefficient analysis but not in the multiple regression analysis

Table 4 shows the results of the correlation analysis between CFAR and sCr, eGFR, and GSR in the zero-hour and IgA groups. No significant relationship was observed between the CFAR and sCr, eGFR, or GSR in the zero-hour group in either the Spearman’s rank correlation coefficient or multiple regression analysis. In the IgA group, CFAR was significantly associated with sCr, eGFR, and GSR in the Spearman’s rank correlation coefficient analysis; however, the multiple regression analysis revealed no significant difference. Figure 5 shows scatter diagrams representing the patients’ CFARs versus sCr, eGFR, and GSR. These results suggest that patient age is an independent factor for increased CFAR.

**Table 4:** Results of correlation analysis between central fibrous area rate (CFAR) and serum creatinine (sCr), estimated glomerular filtration rate (eGFR), and glomerular sclerosis rate (GSR)

	Spearman, two-tailed		Multiple regression analysis
	*r* value	*p* value	*p* value
Zero-hour group			
sCr	0.1477	0.2944	0.1829
eGFR	0.0362	0.9929	0.0533
GSR	0.0675	0.46	0.7843
IgA group			
sCr	0.5343	0.0058	0.1846
eGFR	0.3644	0.0013	0.2646
GSR	0.5697	0.0069	0.4875

## Discussion

This is the first study to quantitatively measure CFAs using renal biopsy specimens. We aimed to verify the hypothesis that renal tissue aging promotes CFA formation. To achieve this aim, we quantitatively measured CFARs in each renal biopsy specimen and statistically evaluated the relationship between patient age and CFAR. The results of the study revealed that older kidney tissue exhibited CFAs more frequently than younger kidney tissue in both the zero-hour and IgA groups. Furthermore, CFAR increased with age more prominently in the IgA group than in the zero-hour group.

Structural and functional changes in kidney tissue related to healthy aging have been observed in various ways. GFR gradually decreases during healthy aging [3, 5, 6, 8]. Studies of relatively healthy people have reported that GFR decreases by 0.4–0.75 ml/min/1.73 m^2^/year [5, 8], or by 6.3–10 ml/min/1.73 m^2^/decade [1, 3]. We also observed that older donors exhibited lower eGFR (decreased by approximately 0.45 ml/min/year), reflective of the healthy aging process.

Some reports support the notion that the glomerular volume increases with healthy aging [1, 2, 4, 9], but other studies have yielded contrasting results [3, 8]. Changes in nephron structure occur during aging [1–3, 8], and aged kidneys appear to exhibit tubule enlargement [3, 8]. The average percentage of connective tissue in non-sclerosed glomeruli increases during aging [1], and podocytes also undergo age-related changes [2, 3]. Researchers have hypothesized that the progressive reduction of viable and normally functioning podocytes leads to glomerular obsolescence and deterioration of the integrity of the slit pore membrane in glomeruli [3]. Hodgin et al. observed that the total glomerular volume and glomerular volume per podocyte increase with age [2]. In addition, some specific podocyte stress and podocytopathy are observed more frequently in aged kidneys. These findings indicate that podocytopathy may be a significant indicator of kidney aging. However, the relationship between the severity of podocytopathy and renal disease remains unclear.

Few studies have evaluated age-related changes in glomerular vascular pole lesions. In 1986, Inomata investigated kidney biopsy specimens and observed globular hyaline deposits around the glomerular vascular pole, referring to these lesions as VPDs [7].

Inomata formed two hypotheses regarding VPD generation. One hypothesis is that plasma proteinaceous materials penetrate the vascular intima of the glomerular vascular pole lesion and stabilize in the local vascular wall. The other is that mesangial deposits in the glomeruli move to the vascular pole lesion via the mesangial channel and accumulate there. However, the author reported no correlation between VPD formation and immune reactions. Indeed, the entire biopsy specimen investigated exhibited some form of renal disease, so it is possible that the VPDs may have been affected by renal diseases.

**Fig. 6:**
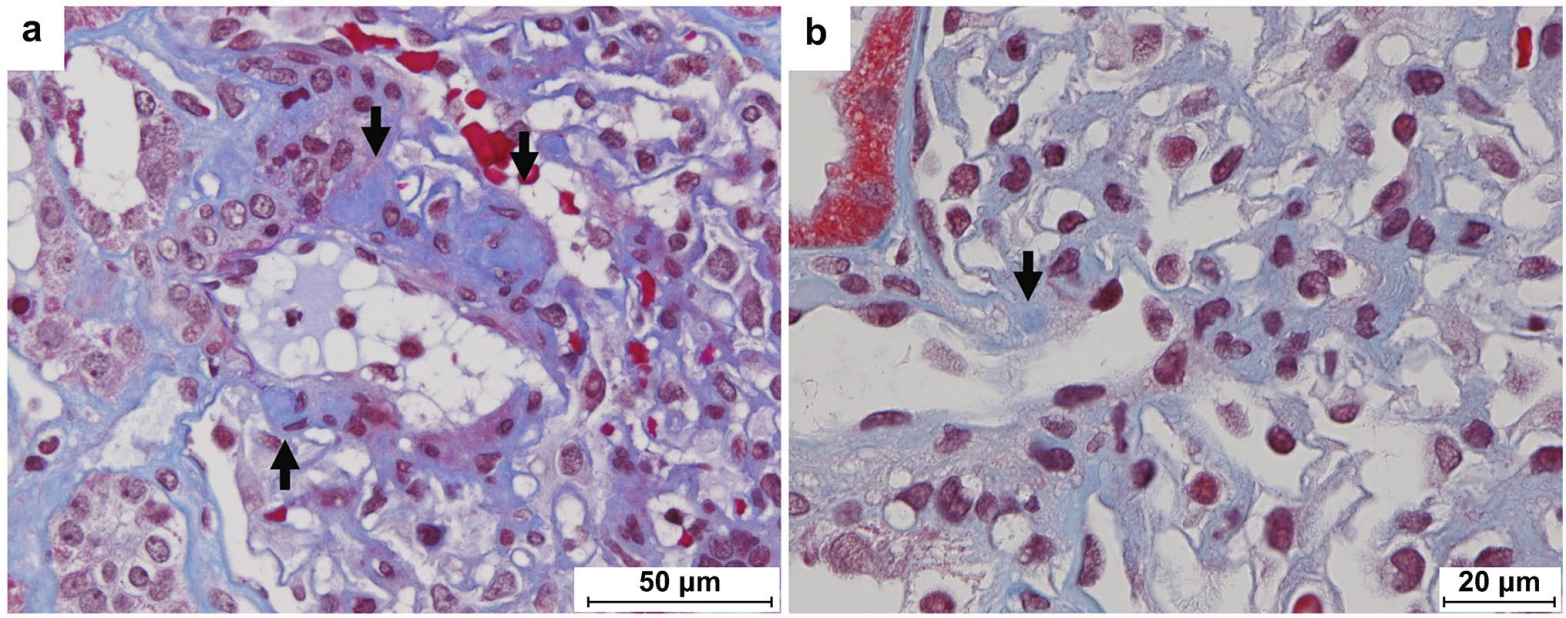
Central fibrous areas (CFAs) from young and old kidneys (zero-hour group). **a** Glomerular vascular pole from a 74-year-old donor kidney (scale bar: 50 μm). A large, apparent CFA is observed (arrow). **b** A glomerular vascular pole from a 40-year-old donor kidney (scale bar 20 μm). The CFA is small and unclear (arrow)

In this study, we observed that CFAs were more frequent and larger in older kidneys than in younger kidneys (Fig. 6). Based on these observations, we hypothesized that CFAs develop during aging. Therefore, we investigated the relationship between CFAs and kidney age using renal transplant donor tissues. Unfortunately, we did not measure the size of the CFAs in this study, although we believe that this is an important parameter that should be investigated in future studies.

For zero-hour donor kidneys, we observed a significant positive correlation between age and GSR; however, for IgA nephropathy kidneys, we observed no significant correlation between patient age and GSR. It is possible that glomerulosclerosis due to IgA nephropathy caused variations in the patients’ GSR, which may have appeared to be a change due to aging. However, we found that for older patients, the CFAR was significantly higher in both the zero-hour and IgA groups. In contrast to the zero-hour group, we observed a positive relationship between patient age and CFAR in the IgA group, and the CFAR of the IgA group exhibited a significantly more rapid increase according to age than the CFAR of the zero-hour group.

The mechanism of CFA formation has rarely been investigated. However, it is known that glomerular capillaries, including vascular pole lesions, are exposed to various types of pressure and movement over time. Glomeruli withstand vascular hydrostatic pressure (60–65 mmHg), plasma colloid osmotic pressure (25 mmHg at the afferent end and up to 35 mmHg at the efferent end), and urinary space hydrostatic pressure (20–25 mmHg) [10]. We presume that persistently high pressure impacting glomerular vascular pole lesions plays a role in CFA formation.

Neal et al. investigated the three-dimensional structure of adult human glomerular capillaries [10]. They found that glomerular vessels at vascular pole lesions expand to a greater extent than connecting arteries, which are called afferent and efferent vascular chambers (VCs). These VC structures have not been observed in either rodent or infant kidneys, in which glomeruli are smaller than adult human glomeruli, which suggests that VCs are associated with large adult glomeruli. These authors simulated hemodynamics in the glomeruli and hypothesized that VCs play a role in ensuring uniform blood distribution to each of the glomerular lobules.

As we observed, a CFA is a structural change that occurs in the VC wall, potentially due to degradation of VC wall components from being exposed to a high-pressure environment for decades, resulting in CFA formation in aged glomeruli. IgA nephropathy is a form of glomerulonephritis with IgA-dominant immunocomplex deposition in the glomerular mesangial and paramesangial lesions. Many IgA nephropathy kidney tissues exhibit proliferative endocapillary changes, crescent formation, and tubulointerstitial damage, in addition to mesangial matrix expansion and mesangial cell proliferation. These structural changes in glomeruli may disturb glomerular blood flow in patients with IgA nephropathy. However, no studies have discussed actual glomerular blood flow in the context of IgA nephropathy. Future studies should examine the relationships among glomerular structure, glomerular blood flow, IgA nephropathy, and CFA formation.

Therefore, it remains to be determined whether IgA nephropathy is associated with renal aging. The Oxford classification of IgA nephropathy is widely used as a histopathological indicator to predict the clinical outcomes of patients with IgA nephropathy [11]. The classification includes histopathological criteria, interstitial fibrosis/tubular atrophy, and arteriosclerosis, and these criteria are also recognized as aging-related changes. Research has demonstrated that patients with IgA nephropathy who are estimated to have more aging-related changes exhibit poorer clinical outcomes, such as older age [12, 13], higher serum creatinine levels, interstitial fibrosis, and tubular atrophy [13].

Kidney tissue with IgA nephropathy may promote age-related or aging-like changes. For example, klotho is a transmembrane protein that acts as a coreceptor for the major phosphatonin, fibroblast growth factor-23 (FGF23), which is regarded as an anti-aging protein [5, 14, 15]. Klotho has various roles in ameliorating vascular calcification, tissue hypoxia, and kidney fibrosis [5, 14]. One study involving a cohort of healthy people aged 70–79 years divided the participants according to their soluble klotho levels, reporting that higher klotho levels were associated with lower odds of relative decreases in kidney function [14]. In contrast, klotho expression in renal tissue affected by IgA nephropathy decreased as its histopathological severity progressed [15]. Indeed, we do not have a clear answer regarding the relationship between IgA nephropathy and tissue aging.

### Limitations

There are some limitations to the current study. First, the influence of renal diseases other than IgA nephropathy on CFA formation is unknown. In this study, wedge biopsies were performed on donor kidneys during bench surgery, and needle biopsies were performed on patients in the departments of nephrology and pediatric nephrology. Therefore, all IgA group kidney specimens, except for six that were changed from the zero-hour group to the IgA group, were obtained via needle biopsy (61/67, 91.0%). This should have caused differences in the total number of glomeruli and total number of glomeruli with global sclerosis found in the zero-hour and IgA groups. The number of observable vascular pole lesions, which we were able to investigate through biopsy specimens (approximately 40% of the total number of glomeruli), was particularly small in the IgA group, causing the CFAR of each biopsy specimen to be scattered. Furthermore, the zero-hour group consisted of adult living donors, while the IgA group included patients of any age who underwent renal biopsy. Therefore, we could not match demographic factors between the zero-hour and IgA groups because of the limited number of patients. As a result of this difference, the zero-hour group was older than the IgA group, and fundamental data such as eGFR and CFAR reflected the difference in age between the two groups.

## Conclusion

The present study demonstrated that aging is an independent factor that promotes CFA formation. In both the zero-hour and IgA groups, biopsy specimens from older patients exhibited significantly higher CFARs, and the IgA group exhibited greater increases in CFAR with age than the zero-hour group. On the other hand, GSR was the most significant aging marker in the zero-hour group; however, there was no relationship between GSR and patient age in the IgA group. These findings indicate that CFA may be a useful indicator of kidney aging, especially in kidneys affected by IgA nephropathy. Nonetheless, further investigation is required to clarify its usefulness.

## Data Availability

The datasets generated and analysed during the current study are available from the corresponding author on reasonable request.
